# Changes in PSA-Based Early Detection of Prostate Cancer over a 12-Year Period: Findings from the German KABOT Study

**DOI:** 10.3390/healthcare14060747

**Published:** 2026-03-16

**Authors:** Kay-Patrick Braun, Torsten Vogel, Matthias May, Christian Gilfrich, Markus Herrmann, Anton P. Kravchuk, Julia Maurer, Ingmar Wolff

**Affiliations:** 1 Institute of General Practice and Family Medicine, Otto von Guericke University Magdeburg, 39120 Magdeburg, Germany; markus.herrmann@med.ovgu.de; 2 MVZ Dr. Braun GmbH, 03046 Cottbus, Germany; 3 General Practice, 16321 Bernau, Germany; t.vogel@arztpraxis-vogel.de; 4 Department of Urology, Brothers of Mercy Hospital, Straubing, 94315 Straubing, Germany; matthias.may@klinikum-straubing.de (M.M.); christian.gilfrich@klinikum-straubing.de (C.G.);; 5 University Cancer Center UCC-R, University Hospital Regensburg, 93053 Regensburg, Germany; 6 Department of Urology, University Medicine Greifswald, 17475 Greifswald, Germany

**Keywords:** health literacy, preventive health behavior, socioeconomic determinants, smoking status, primary care

## Abstract

**Background:** The effectiveness of prostate-specific antigen (PSA)-based early detection of prostate cancer remains controversial and implementation-dependent. Screening policy changes have substantially altered PSA testing behavior in the United States, yet longitudinal evidence from non-organized European settings is limited. We assessed 12-year changes in awareness and utilization of PSA-based early detection and identified subgroups requiring targeted counseling. **Methods:** Two cross-sectional survey waves were conducted in 2009 (Study Phase 1) and 2021 (Study Phase 2) among men recruited via general practitioner practices in urban and rural regions of Germany. The survey was developed and reported according to the Consensus-Based Checklist for Reporting of Survey Studies (CROSS). Identical questionnaires were used across phases. Endpoints were awareness of PSA-based early detection and prior PSA testing. Univariable and multivariable logistic regression evaluated independent associations with sociodemographic and behavioral factors. To assess sensitivity to compositional differences between survey waves, post-stratified weighted analyses re-aligning Study Phase 2 to the Study Phase 1 distribution of age category, educational attainment, and smoking status were conducted. **Results:** The analytic cohort comprised 890 men (Study Phase 1, n = 755; Study Phase 2, n = 135). Compared with Study Phase 1, Study Phase 2 participants more frequently were non-smokers (63.0% vs. 48.5%, *p* < 0.001) and had a university degree (38.5% vs. 30.5%, *p* = 0.002). In primary multivariable analyses, higher educational attainment (OR 1.71, 95% CI 1.24–2.36) and paternity (OR 1.94, 95% CI 1.25–3.01) were independently associated with greater awareness, whereas increasing age (OR 1.39, 95% CI 1.29–1.50) and higher educational attainment (OR 1.63, 95% CI 1.19–2.24) were independently associated with utilization. Study phase was not independently associated with either endpoint in primary models. In post-stratified sensitivity analyses, study phase was positively associated with utilization, indicating sensitivity of temporal contrasts to population composition. **Conclusions:** In primary multivariable analyses, we did not detect statistically significant temporal differences in awareness or utilization of PSA-based early detection within this German non-organized setting. The emergence of a study phase effect in weighted sensitivity analyses suggests that apparent time trends may be influenced by compositional differences between survey waves. Persistent social gradients, particularly related to educational attainment, underscore the importance of targeted, evidence-based counseling in opportunistic early detection systems. Larger, prospectively designed studies are needed to distinguish true temporal change from sampling-related effects.

## 1. Introduction

Prostate cancer is the most frequently diagnosed malignancy among men in Germany and the United States, with approximately 65,820 and 268,500 newly diagnosed cases per year, respectively [1,2]. It represents the second leading cause of cancer-related death in men in both countries, accounting for 15,379 deaths annually in Germany and 34,500 in the United States [1,2]. Incidence rates peak in men in their eighth decade of life, whereas prostate cancer mortality is highest among men aged 80 years and older [2,3]. Globally, prostate cancer remains the most common cancer and the fifth leading cause of cancer-related death among men, with an estimated 1.4 million new cases and 375,000 deaths reported worldwide in 2020 [1,2,3].

Marked disparities in prostate cancer incidence and outcomes have been consistently observed. African American men exhibit the highest incidence rates worldwide [4]. A positive family history constitutes another well-established, non-modifiable risk factor [4]. Several potentially modifiable factors have also been associated with prostate cancer incidence, including high alcohol consumption, dietary calcium and milk protein intake, and hormonal influences, particularly involving testosterone and dihydrotestosterone [4,5,6]. In contrast, higher consumption of tomatoes, increased physical activity, intake of phytoestrogens such as those found in soy products, higher coffee consumption, and greater sexual activity have been discussed as protective factors [7,8,9,10,11]. Notably, current smoking status has repeatedly been associated with a lower reported incidence of prostate cancer [12]. This paradoxical finding has been attributed to lower participation in prostate cancer early detection among smokers rather than a true protective effect. Large population-based data have demonstrated that active smokers are approximately one-third less likely to undergo prostate-specific antigen testing compared with non-smokers, even after multivariable adjustment [13]. Importantly, despite lower detection rates, smokers exhibit a significantly increased risk of prostate cancer-specific mortality [14].

Prostate-specific antigen testing was introduced in 1994 as an adjunct to digital rectal examination for the early detection of prostate cancer [15]. Potential harms of PSA-based early detection include complications related to subsequent prostate biopsy as well as the risks and long-term consequences of overtreatment [16]. Although the effectiveness of PSA-based early detection remains controversial, robust evidence supports a reduction in prostate cancer-specific mortality in defined age groups. The European Randomized Study of Screening for Prostate Cancer demonstrated a 27% reduction in cancer-specific mortality after a median follow-up of 21 years, despite substantial non-compliance and contamination in both study arms [17]. Conversely, population-level data from the United States indicate that the discontinuation of routine PSA-based screening has been accompanied by an unfavorable stage migration. While incidence rates among men aged 45 to 74 years remained stable between 2004 and 2010, a significant increase in metastatic prostate cancer was observed from 2010 onward following changes in screening recommendations [18].

In 2012, the United States Preventive Services Task Force recommended against routine PSA-based screening [19]. This position was revised in 2018, endorsing shared decision-making for men aged 55 to 69 years [20]. Subsequent studies from the United States consistently reported a sustained decline in PSA testing following these changes, accompanied by reductions in prostate biopsy rates, prostate cancer incidence, and the use of definitive local therapies [21,22,23]. These findings highlight the sensitivity of early detection practices to guideline recommendations and health policy decisions.

In Germany, early detection of prostate cancer is performed by both urologists and general practitioners and is offered from the age of 45 years. Men with statutory health insurance are entitled to an annual digital rectal examination; however, this examination does not constitute a screening test and has limited sensitivity for early-stage disease [24]. Measurement of PSA levels is not routinely reimbursed and is performed only upon patient request after counseling regarding potential benefits and harms [25]. Consequently, PSA-based early detection in Germany largely depends on individual awareness, physician counseling, and patient-driven decision-making.

The effectiveness of any screening strategy depends not only on its diagnostic performance but also on its implementation within the target population. Beyond age, sex, and ethnicity, several patient-related factors, including educational attainment, income, insurance status, and marital status, have been shown to substantially influence screening behavior [21,22,26,27,28,29,30,31,32,33,34]. Recent epidemiological studies have further demonstrated that PSA testing uptake is closely linked to broader health behaviors and indicators of preventive care engagement, underscoring the complex interplay between sociodemographic factors and early detection practices [35].

In parallel with the ongoing controversy surrounding PSA-based early detection, European urological initiatives have increasingly shifted the focus from test-based screening towards structured, risk-adapted early-detection pathways [36]. These concepts aim to preserve potential mortality benefits while reducing harms through improved pre-biopsy risk stratification, integration of magnetic resonance imaging, and consistent use of active surveillance for low-risk disease [36,37,38]. Within this framework, digital rectal examination is no longer considered a stand-alone screening tool, but rather an adjunctive clinical assessment with limited discriminatory value when used in isolation [37]. Similarly to population-based breast cancer screening, the emerging consensus emphasizes that the value of early detection depends less on the screening test itself than on the quality, structure, and governance of the diagnostic pathway in which it is embedded [36].

Throughout this manuscript, we use the term “PSA-based early detection” to refer to PSA testing undertaken in asymptomatic men with the intent to detect prostate cancer at an earlier stage, acknowledging that this occurs in an opportunistic, non-organized setting in Germany.

Identifying population subgroups with limited awareness or reduced utilization of PSA-based early detection is therefore essential for targeted counseling and informed decision-making. The present study evaluates awareness and utilization of PSA-based early detection of prostate cancer over a 12-year period, enabling an assessment of temporal changes in the context of evolving evidence and international guideline recommendations. Of particular interest is whether the decline in PSA testing observed in the United States following the 2012 guideline change is similarly reflected in a German healthcare setting.

In addition to quantifying utilization patterns, this study assesses knowledge and awareness of PSA-based early detection among men considered eligible for testing. Given the lack of comparable longitudinal studies spanning more than a decade, this pilot study aims to provide a data-driven foundation for future large-scale investigations into early detection behavior and targeted counseling strategies.

## 2. Materials and Methods

### 2.1. Implementation of the Study

In 2009, an interdisciplinary working group consisting of general practitioners and urologists conducted a cross-sectional survey study to assess attitudes, knowledge, and utilization patterns related to prostate cancer early detection based on prostate-specific antigen testing. A structured questionnaire comprising 20 items was developed, refined, and validated through 12 structured individual interviews to ensure clarity, comprehensibility, and content validity.

Study Phase 1 (SP1) was conducted in 2009. A total of 55 general practitioner practices were randomly selected from a database provided by the regional Association of Statutory Health Insurance Physicians in Berlin and Brandenburg. Each participating practice received 50 questionnaires for distribution to male patients aged 35 years or older who were considered capable of autonomously completing the survey.

Study Phase 2 (SP2) was initiated in October 2021 to evaluate temporal changes over a 12-year interval. This follow-up survey was conducted under the study label “Knowledge and Belief Over Time (KABOT)”. The questionnaire content remained largely identical to that of SP1, with the addition of two thematic blocks addressing contemporary aspects of prostate cancer early detection. The moderate response rate observed in SP1 of 38.2% raised concerns regarding sample size and representativeness. To address these limitations, the number of contacted general practitioner practices was increased to 150 in SP2.

Practices in SP2 were selected using an updated random sampling approach and were complemented by practices affiliated with the KV RegioMed teaching network, identified using predefined geographic search criteria (general practitioner practices located in or around Cottbus or Bernau within a 5 km radius) [39,40]. A reminder letter was distributed in January 2022. All questionnaires returned by 31 March 2022, were included in the final analysis. Data collection was performed by physicians accredited within the statutory health insurance system, while exclusively private practices were excluded. All surveys were completed anonymously in full compliance with applicable data protection regulations.

Ethical approval was obtained from the Ethics Committee of the Brandenburg State Medical Association (approval number 2021-2126-BO-ff) on 10 August 2021. The study was registered in the German Registry of Clinical Studies (DRKS registration number 00027862) [41].

No formal a priori power calculation was performed due to the exploratory and observational nature of the study.

### 2.2. Structure of the Questionnaires

**Figure A1:**
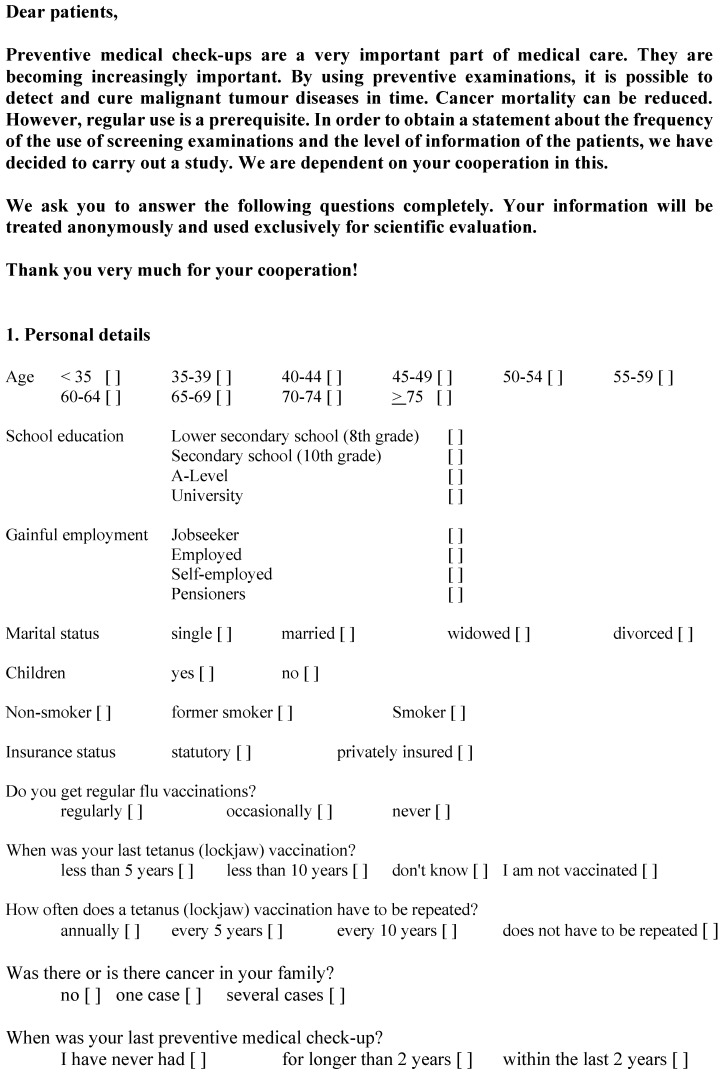

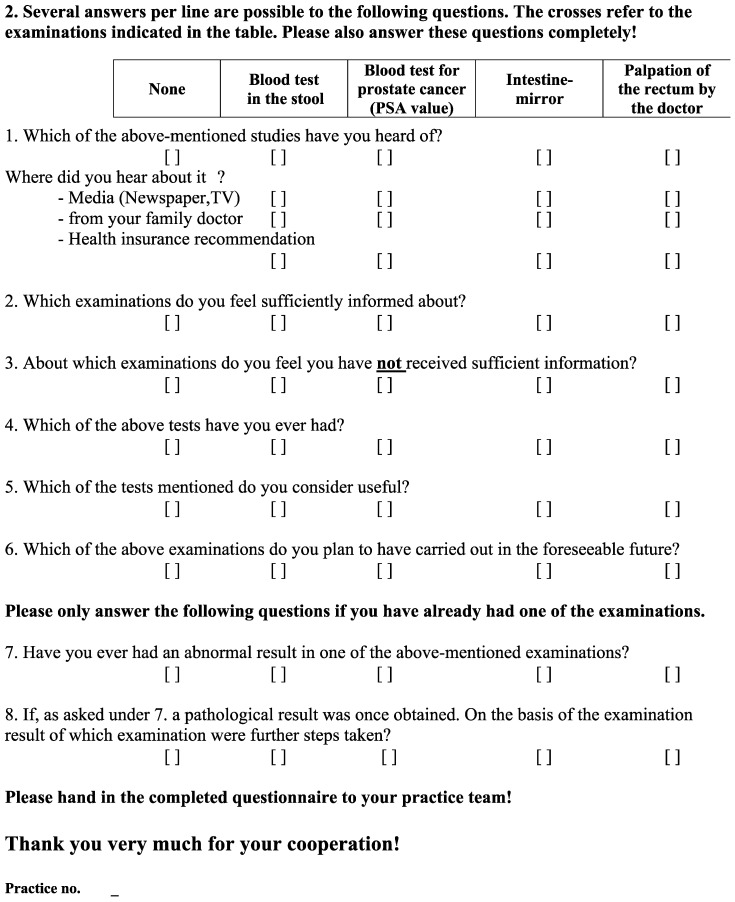
Questionnaire SP1 (original questionnaire in German, translation for publication).

Questionnaire development followed the Consensus-Based Checklist for Reporting of Survey Studies (CROSS), with full adherence documented in Appendix A Table A1 and Table A2 [42]. The patient questionnaire used in SP1 included 11 items addressing sociodemographic characteristics, family history, and vaccination status, as well as nine additional items assessing knowledge, perceptions, and personal utilization of prostate cancer early detection (Figure A1).

**Figure A2:**
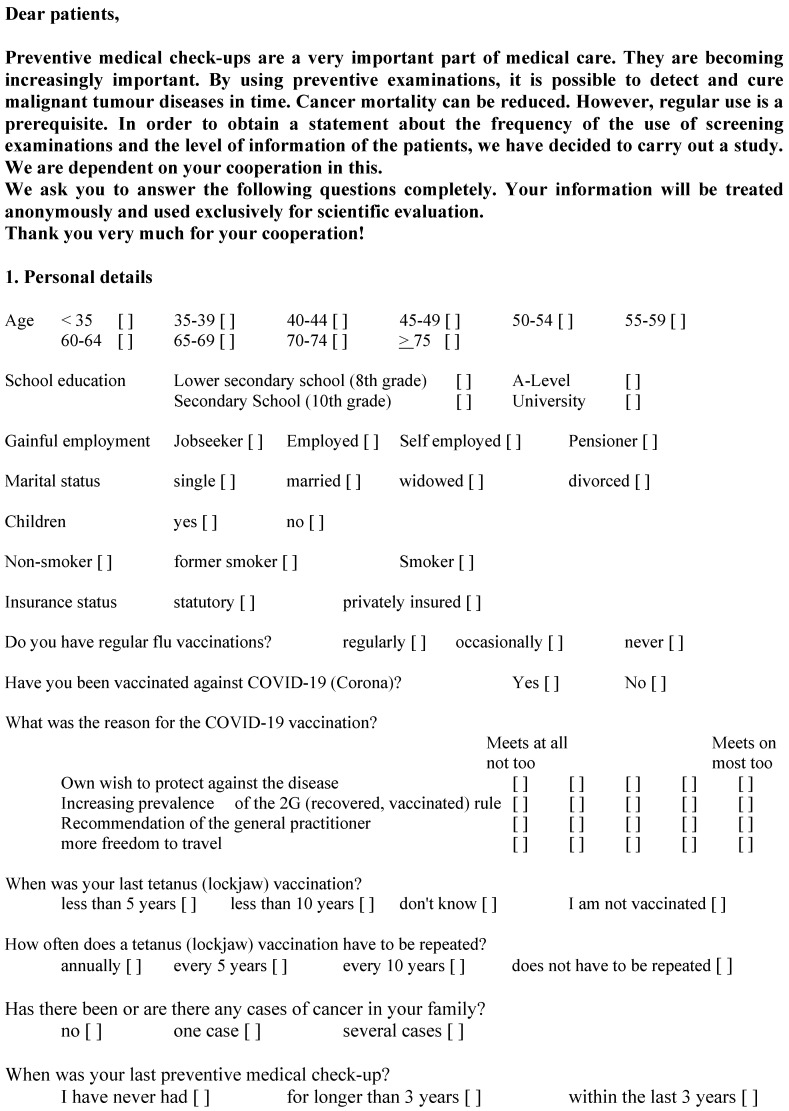

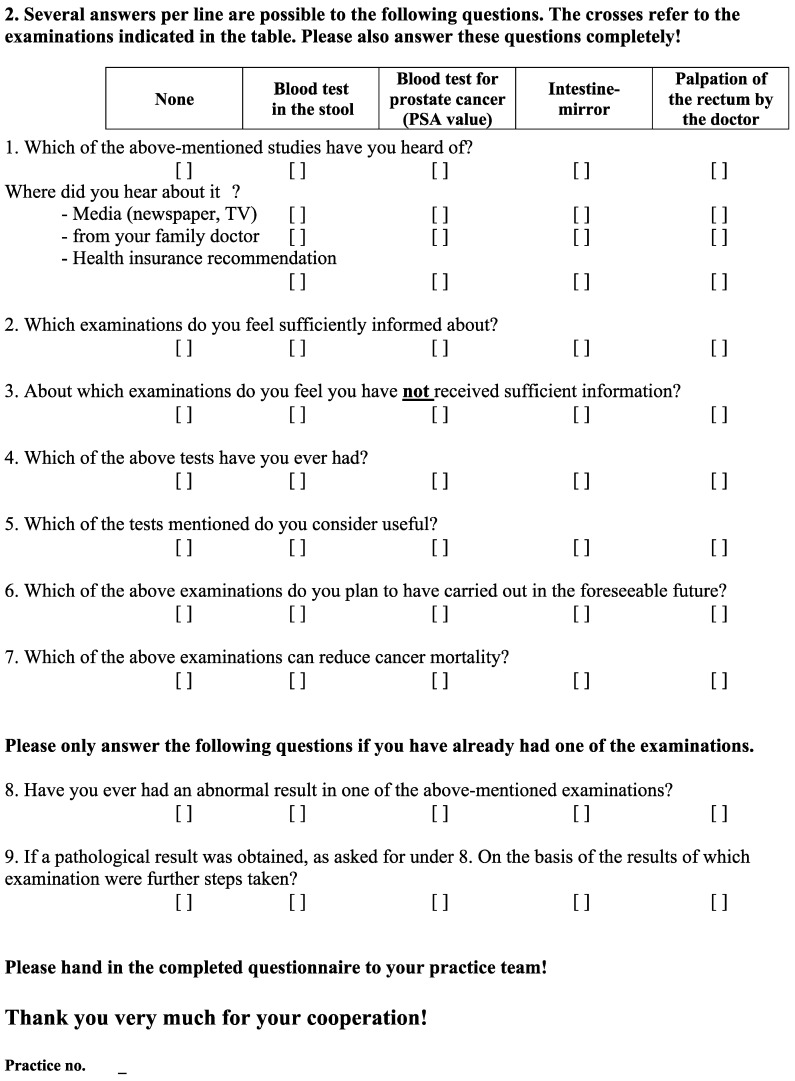
Questionnaire SP2 (original questionnaire in German, translation for publication).

In SP2, one additional item was included to assess participants’ perceptions regarding the potential impact of early detection on cancer-specific mortality (Figure A2). Pilot testing was performed in both study phases with 12 participants each, resulting in minor adjustments to optimize survey length, internal consistency, clarity, and completeness.

All questionnaire items assessing awareness and prior utilization of PSA-based early detection were identical in wording and response options across Study Phase 1 and Study Phase 2.

Response rates were calculated at the practice level and refer to practices that actively participated by returning completed patient questionnaires.

### 2.3. Research Questions and Statistical Methods

The primary objective of this study was to identify factors independently associated with prostate cancer early detection awareness and utilization. For clarity and focus, analyses were restricted to early detection of prostate cancer.

Age was recorded using predefined categorical response options, including a category for participants younger than 35 years, followed by 5-year increments from 35–39 years up to a category of 75 years and older. Smoking status was recorded as non-smoker, former smoker, or current smoker and was dichotomized for regression analyses into “non-smoker or former smoker” as the reference category versus “current smoker”. Educational attainment was assessed using four categories reflecting the German educational system: basic secondary school qualification, secondary school certificate, higher school certificate, and university degree. For analysis, these were dichotomized into “basic secondary school qualification or secondary school certificate” versus “higher school certificate or university degree”.

Family history of cancer was recorded as “none”, “one case”, or “several cases” and subsequently dichotomized into “no family history of cancer” versus “family history of cancer”. Paternity was categorized as “no children” versus “children”. Employment status options included “jobseeker”, “employed”, “self-employed”, and “pensioner”, and were dichotomized into “jobseeker” versus “employed, self-employed, or pensioner”. Insurance status was recorded as statutory or private health insurance. Study phase (SP1 versus SP2) was included as an independent variable in all analyses.

Two predefined binary endpoints were analyzed: awareness of prostate-specific antigen-based early detection and actual utilization of prostate-specific antigen-based early detection. Univariable logistic regression models were first applied to assess the individual association of each independent variable with the endpoints. Subsequently, multivariable logistic regression models were constructed to identify independent predictors while accounting for potential confounding. Variables were included in the multivariable models based on clinical relevance and statistical significance in univariable analyses, with all dichotomized variables entered using the lower-risk or absence category as the reference group. In case of an incompletely answered questionnaire, available answers were nevertheless considered and appropriately reflected in the analysis.

Comparisons of baseline characteristics between SP1 and SP2 were performed using chi-squared tests for categorical variables and independent-samples *t*-tests for continuous variables. Effect estimates are reported as odds ratios with corresponding 95% confidence intervals (95% CI). Statistical significance was defined as a two-sided *p*-value of 0.05 or less.

To assess the sensitivity of temporal associations to compositional differences between survey waves, we conducted a post-stratification analysis. Study Phase 2 observations were re-weighted to match the marginal distribution of age category, educational attainment, and smoking status observed in Study Phase 1. Normalized weights were applied to multivariable logistic regression models for both endpoints. These weighted models were prespecified as exploratory sensitivity analyses to evaluate the robustness of phase-specific associations.

To account for potential intra-practice correlation, we additionally re-estimated all multivariable models using practice-level cluster-robust standard errors. Model performance was assessed using the area under the receiver operating characteristic curve and the Hosmer–Lemeshow goodness-of-fit test. Multicollinearity was evaluated using variance inflation factors.

Item-level missingness is summarized in Table A3; available-case analysis was applied for each model.

All statistical analyses were conducted using SPSS software version 29.0 (IBM Corp., Armonk, NY, USA).

## 3. Results

**Figure 1:**
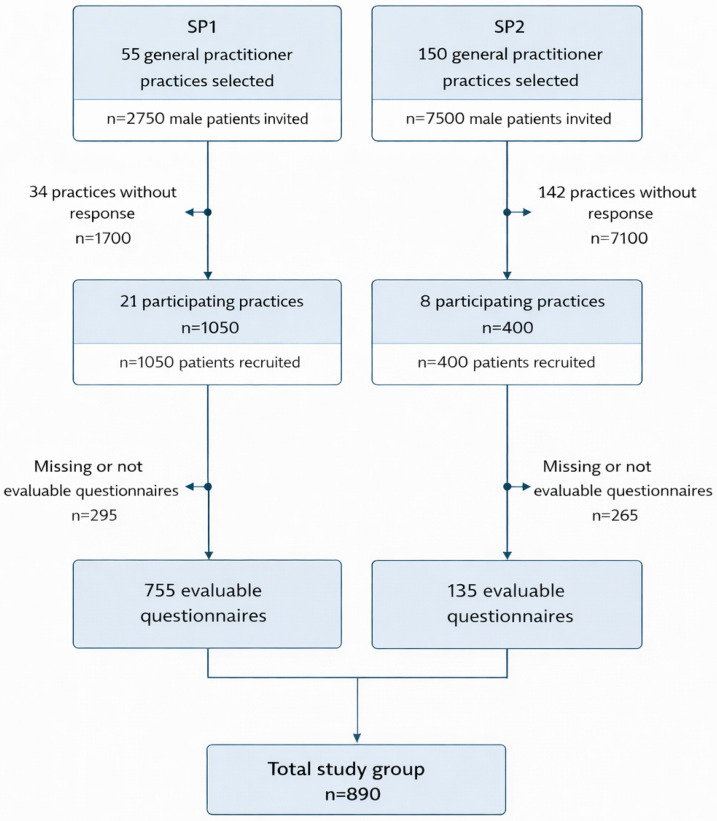
Flow diagram of practice participation and survey response in Study Phase 1 (2009) and Study Phase 2 (2021), including numbers of participating practices, questionnaires distributed and returned, and the analytic sample. *Legend:* Flow chart illustrating the recruitment of general practitioner practices and study participants, as well as the number of evaluable questionnaires included in Study Phase 1 (2009; Response rate 38.2%) and Study Phase 2 (2021; Response rate 5.3%). The figure depicts the sequential selection process and the final analytic cohort used for all statistical analyses.

The composition of the study population across both study phases is illustrated in the flow chart shown in Figure 1. The analytic cohort comprised 755 participants in Study Phase 1 (SP1) and 135 participants in Study Phase 2 (SP2) (total n = 890) (Table 1).

### 3.1. Participant Characteristics

Baseline characteristics stratified by study phase are summarized in Table 1. Compared with SP1, SP2 included a higher proportion of non-smokers (63.0% vs. 48.5%, *p* < 0.001) and a higher proportion of participants with a university degree (38.5% vs. 30.5%, *p* = 0.002). Employment status distributions differed between SP1 and SP2 (*p* < 0.001), with a markedly higher share of self-employed participants in SP2 (45.2% vs. 4.9%). Paternity was less frequent in SP2 (80.0% vs. 85.4%, *p* = 0.019). In contrast, differences by study phase were not statistically significant for age distribution (*p* = 0.068), family history of cancer (*p* = 0.102), or insurance status (*p* = 0.344).

### 3.2. Awareness of PSA-Based Early Detection

Univariable and multivariable logistic regression results for the endpoint “Awareness of PSA-based early detection” are presented in Table 2. In univariable analyses, awareness increased with age (OR 1.063, 95% CI 1.005–1.124; *p* = 0.032) and was higher among participants with higher educational attainment (OR 2.021, 95% CI 1.496–2.730; *p* < 0.001) and among participants with children (OR 2.081, 95% CI 1.414–3.064; *p* < 0.001). Current smoking was significantly associated with lower awareness of PSA-based early detection (OR 0.553, 95% CI 0.397–0.770; *p* < 0.001). Employment status was also associated with awareness (OR 1.829, 95% CI 1.108–3.018; *p* = 0.018). Study phase was not associated with awareness in univariable analysis (OR 1.028, 95% CI 0.699–1.513; *p* = 0.888).

In the multivariable model, higher educational attainment (OR 1.709, 95% CI 1.239–2.357; *p* = 0.001), smoking status (OR 0.688, 95% CI 0.479–0.989; *p* = 0.043), and paternity (OR 1.939, 95% CI 1.249–3.012; *p* = 0.003) remained independently associated with awareness, whereas age (OR 0.990, 95% CI 0.926–1.058; *p* = 0.766) and employment status (OR 1.528, 95% CI 0.888–2.631; *p* = 0.126) were no longer significant after adjustment. Family history of cancer and insurance status were not independently associated with awareness.

In addition, Table A4 shows the multivariable analysis taking into account educational attainment coded in four levels and smoking status coded as non-smoker, former and current smoker.

The post-stratified sensitivity analyses, in which the weighting of Study Phase 2 was applied to the distribution of age categories, educational levels, and smoking status in Study Phase 1, are presented in Table A5.

### 3.3. Utilization of PSA-Based Early Detection

Regression results for the endpoint “Already underwent PSA-based early detection” are summarized in Table 3. In univariable analyses, utilization was higher in SP2 than in SP1 (OR 1.476, 95% CI 1.022–2.131; *p* = 0.038) and increased with age (OR 1.417, 95% CI 1.327–1.513; *p* < 0.001). Higher educational attainment (OR 1.873, 95% CI 1.422–2.469; *p* < 0.001), paternity (OR 2.646, 95% CI 1.718–4.076; *p* < 0.001), and employment status (OR 2.473, 95% CI 1.402–4.362; *p* = 0.002) were positively associated with utilization, whereas smoking was inversely associated (OR 0.378, 95% CI 0.264–0.541; *p* < 0.001).

In the multivariable model, increasing age (OR 1.391, 95% CI 1.291–1.498; *p* < 0.001) and higher educational attainment (OR 1.632, 95% CI 1.190–2.239; *p* = 0.002) remained positively associated with utilization, while current smoking remained independently associated with reduced utilization of PSA-based early detection (OR 0.608, 95% CI 0.405–0.912; *p* = 0.016). Study phase was no longer significant after adjustment (OR 1.365, 95% CI 0.885–2.105; *p* = 0.160). Paternity and employment status also lost statistical significance in the multivariable model.

In addition, Table A6 shows the multivariable analysis taking into account educational attainment coded in four levels and smoking status coded as non-smoker, former and current smoker.

In post-stratified sensitivity analyses applying re-weighting of Study Phase 2 to the Study Phase 1 distribution of age category, educational attainment, and smoking status, study phase was positively associated with utilization. Full model estimates are provided in Table A7.

### 3.4. Subgroup Analysis in Men Aged 45–69 Years

Subgroup analyses restricted to men aged 45–69 years are shown in Table 4 and Table 5. For awareness of PSA-based early detection (Table 4), higher educational attainment was associated with awareness in univariable analysis (OR 1.875, 95% CI 1.258–2.794; *p* = 0.002) and remained significant after adjustment (OR 1.717, 95% CI 1.113–2.647; *p* = 0.014). Current smoking was independently associated with lower awareness of PSA-based early detection in both univariable (OR 0.525, 95% CI 0.343–0.804; *p* = 0.003) and multivariable analyses (OR 0.595, 95% CI 0.375–0.945; *p* = 0.028). Study phase was not associated with awareness in this subgroup.

For utilization of PSA-based early detection in men aged 45–69 years (Table 5), age was strongly associated with utilization in univariable analysis (OR 1.528, 95% CI 1.338–1.745; *p* < 0.001) and remained significant in the adjusted model (OR 1.520, 95% CI 1.315–1.757; *p* < 0.001). Higher educational attainment was also independently associated (OR 1.590, 95% CI 1.077–2.347; *p* = 0.020). Current smoking remained associated with a lower likelihood of utilization in the adjusted model, reaching borderline statistical significance (OR 0.627, 95% CI 0.393–1.001; *p* = 0.051). Smoking showed a borderline association after adjustment (OR 0.627, 95% CI 0.393–1.001; *p* = 0.051). Study phase, employment status, family history of cancer, and insurance status were not independently associated with utilization in this subgroup.

### 3.5. Cross-Table Analyses

The attenuation of unadjusted differences after multivariable adjustment suggests confounding by compositional differences rather than true temporal change.

**Figure A3:**
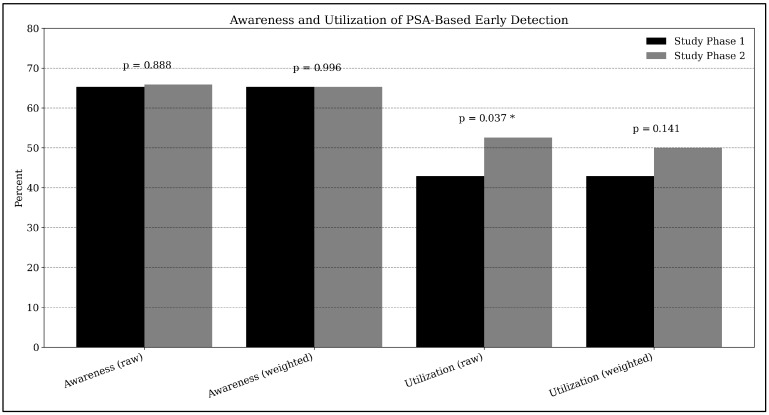
Side-by-side bar plots of awareness of PSA-based early detection and prior utilization of PSA testing in Study Phase 1 and Study Phase 2. “Raw” bars reflect observed proportions. “Weighted” bars reflect SP2 proportions post-stratified to the SP1 distribution of age category, educational attainment, and smoking status. (* statistically significant).

In visual analyses using post-stratified standardization, crude phase differences attenuated, and no meaningful differences in standardized proportions were observed (Figure A3).

Across all multivariable models, study phase, employment status, family history of cancer, and insurance status were not independently associated with either endpoint (Table 2, Table 3, Table 4 and Table 5).

In addition, the practice-level cluster-robust standard errors in multivariable models, the variance inflation factor, and the Receiver Operating Characteristics (ROC) analysis for awareness and utilization of PSA-based early detection are presented in Table A8, Table A9 and Table A10.

## 4. Discussion

This longitudinal survey study provides several novel insights into prostate-specific antigen-based early detection behavior in a non-organized screening setting over a 12-year period. First, despite substantial changes in international screening recommendations and accumulating evidence on the benefits and harms of PSA-based early detection, in primary multivariable analyses, we did not detect statistically significant temporal variations in utilization patterns in Germany. Second, no change over time was observed in terms of pronounced social gradients, with educational attainment and smoking behavior emerging as the most consistent determinants of both awareness and utilization of PSA-based early detection. Third, increasing age was strongly associated with utilization but not with awareness, indicating a widening gap between knowledge and action across the life course. Together, these findings add a longitudinal, population-based perspective to the international literature and highlight persistent structural determinants of early detection behavior.

The absence of a measurable study phase effect contrasts sharply with data from the United States, where the 2012 recommendation of the U.S. Preventive Services Task Force against routine PSA screening was followed by a sustained decline in PSA testing across all age groups [21,22,43,44,45,46,47,48,49]. Several studies have further demonstrated downstream consequences, including reductions in prostate biopsies, prostate cancer incidence, and definitive local therapy [23], accompanied by a subsequent increase in advanced and metastatic disease [18,47]. In particular, Desai et al. reported a significant rise in metastatic prostate cancer among men aged 45–74 years after 2011, whereas incidence rates had remained stable between 2004 and 2010 [18]. In contrast, our data indicate that these international developments did not translate into reduced PSA-based early detection use in Germany. This discrepancy likely reflects fundamental differences in health care organization, reimbursement structures, and the absence of centralized screening policies in Germany, where PSA testing remains largely patient-driven and dependent on individual counseling. The population-level increase in metastatic prostate cancer reported by Desai et al. occurred in the context of centrally influential USPSTF recommendations within an insurance-based healthcare system and should therefore not be directly extrapolated to opportunistic, non-organized screening environments such as Germany [18].

**Figure A4:**
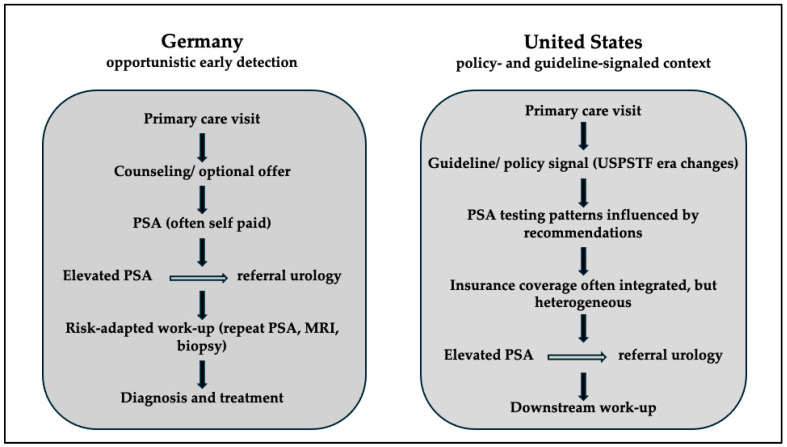
Conceptual schematic contrasting the opportunistic German PSA-based early-detection pathway with a guideline- and policy-signaled context exemplified by the United States. The figure highlights differences in governance signals and access conditions that may influence population testing behavior.

To aid interpretation, we provide a schematic contrast of the opportunistic German early-detection pathway, in which PSA testing is typically offered following individual counseling and is often self-paid, versus a policy- and guideline-signaled environment such as the United States, where changes in national recommendations have been associated with measurable shifts in PSA testing patterns (Figure A4).

Our findings of not statistically significant changes in awareness and utilization of PSA-based early detection over a 12-year period should be interpreted in the context of rapidly evolving European early-detection concepts led by urologic stakeholders [36,37,38]. Recent European initiatives emphasize that PSA testing alone is insufficient to generate population-level value and must be embedded within structured, risk-adapted algorithms incorporating downstream triage tools such as magnetic resonance imaging and defined surveillance strategies [36,38]. This paradigm mirrors the experience from population-based mammography screening, where quality assurance, standardized pathways, and centralized governance were essential to balance benefits and harms despite persistent debate on overdiagnosis [36]. In contrast, the opportunistic nature of PSA testing in Germany may partly explain why evolving evidence and recommendations have not translated into measurable changes in early-detection behavior within the present study.

Educational attainment emerged as the most robust determinant of PSA-based early detection behavior in this study. Higher education was independently associated with both awareness and utilization, in the overall cohort as well as in men aged 45–69 years. These findings are consistent with prior studies demonstrating higher PSA testing rates among men with higher educational levels [28]. At the same time, conflicting evidence exists. Pickles et al. reported higher PSA testing rates among men with lower educational attainment and limited health literacy, attributing this pattern to insufficient understanding of overdiagnosis and overtreatment [32]. Taken together, these findings underscore that educational level does not uniformly translate into more appropriate screening decisions, but rather modulates how information is processed and acted upon. From a public health perspective, our results suggest that men with lower educational attainment represent a key target group for structured, comprehensible counseling that facilitates informed and self-determined decision-making.

Smoking status constituted another consistent determinant of PSA-based early detection behavior. In both the overall cohort and the age-restricted subgroup, smokers showed significantly lower awareness and utilization of PSA-based early detection, with smoking remaining an independent predictor after multivariable adjustment. This finding aligns closely with population-based data from Golijanin et al., who demonstrated that active smoking was associated with approximately one-third lower odds of PSA testing among more than 50,000 screening-eligible men [27]. These behavioral patterns also provide a plausible explanation for epidemiological observations linking smoking to a lower reported incidence of prostate cancer during the PSA screening era [12], despite a clearly increased risk of prostate cancer-specific mortality among smokers [14]. Together, these data indicate that reduced engagement in preventive health behavior, rather than biological protection, underlies this association and identifies smokers as a particularly vulnerable group for targeted counseling interventions.

Paternity was associated with higher awareness of PSA-based early detection but did not translate into higher utilization after adjustment. This dissociation may reflect greater general health awareness among men with children, without a corresponding increase in decisive action. Similarly, age showed a strong and independent association with utilization but not with awareness. This pattern suggests that while knowledge about PSA-based early detection remains relatively stable across age groups, actual uptake increases with advancing age. This finding is particularly relevant given robust evidence from the ERSPC demonstrating a reduction in prostate cancer-specific mortality in men aged 50–65 years [17]. It underscores the need to ensure that counseling is not deferred until older age, but rather reaches men at a time when the potential benefit of early detection is greatest.

In post-stratified sensitivity analyses aligning Study Phase 2 to the Study Phase 1 distribution with respect to age category, educational attainment, and smoking status, study phase was positively associated with utilization. This shift suggests that crude phase contrasts are sensitive to compositional differences between survey waves. However, given the exploratory nature of the weighting procedure and the limited number of participants in Study Phase 2, these findings should be interpreted cautiously and considered hypothesis-generating.

A range of additional determinants of PSA-based early detection behavior have been described in the literature, including marital status, income, insurance status, and family history of cancer. An overview of selected findings is provided in Table 6.

In contrast to some prior reports [21,22,26,27,28,29,30,31,32,33,34], we did not observe an independent association of family history of cancer or employment status with awareness or utilization of PSA-based early detection. Insurance status was likewise not associated with PSA testing behavior, despite the fact that PSA testing is not routinely reimbursed by statutory health insurance in Germany but is commonly covered by private insurance. These findings suggest that financial coverage alone may not be sufficient to overcome informational and behavioral barriers to early detection.

An important contextual factor underlying our findings is the opportunistic nature of PSA-based early detection in Germany. In contrast to organized, population-based screening programs, PSA testing is not reimbursed as a preventive service and is typically offered as a self-paid examination following individual counseling. As a result, access to PSA-based early detection is inherently shaped by socioeconomic resources, health literacy, and patients’ willingness and ability to bear out-of-pocket costs.

Within this framework, the pronounced associations with educational attainment and smoking behavior likely reflect structural access gradients rather than individual risk perception alone. Men with higher educational levels may be more familiar with preventive health concepts and better positioned to navigate discretionary testing, whereas current smokers and individuals with lower educational attainment may face cumulative barriers to engagement. These system-level characteristics provide a plausible explanation for the persistent social gradients observed across both study phases and underscore the importance of interpreting PSA utilization patterns within their specific health system context.

The strengths of this study include its extended observation period of 12 years, the assessment of both awareness and actual utilization of PSA-based early detection, and the use of consistent methodology across study phases. To our knowledge, no comparable longitudinal survey addressing these aspects over such a time span has been published to date. Several limitations must nevertheless be acknowledged. The most important limitation is the relatively small sample size in Study Phase 2, which coincided with the coronavirus pandemic and may have affected participation. The smaller sample size in Study Phase 2 limits statistical power for detecting modest temporal effects and may increase the risk of type II error in subgroup analyses. Differences in participant characteristics between SP1 and SP2, particularly with respect to smoking behavior, educational attainment, and employment status, raise concerns regarding cohort comparability. The higher share of self-employed respondents in Study Phase 2 may reflect differential access to PSA-based early detection within the German opportunistic, self-paid screening context. Self-employment may be associated with fewer financial or administrative barriers to discretionary preventive services, emphasizing the role of structural and socioeconomic factors in shaping utilization patterns. In addition, recruitment was limited to patients visiting general practitioner practices, introducing potential selection bias toward individuals with higher health care utilization. The limited number of participating physicians further restricts generalizability, and it remains unclear to what extent participating physicians actively supported early detection and counseling. Given the limited practice participation and the absence of information on non-responders, external validity is constrained, and the findings should be interpreted with appropriate caution. Finally, the questionnaire did not capture the depth or quality of participants’ knowledge regarding potential harms of PSA-based early detection, precluding a more nuanced assessment of informed decision-making.

In summary, this descriptive and hypothesis-generating pilot study demonstrates persistent social gradients in PSA-based early detection behavior over a 12-year period, despite evolving international evidence and recommendations. Educational attainment and smoking status emerged as the most consistent determinants of both awareness and utilization, while no temporal decline in PSA testing was observed in the German non-organized screening setting. These findings underscore the need for targeted, evidence-based counseling strategies that address structural and behavioral barriers to informed participation in early detection. Future implementation research should focus on linking patient knowledge, screening behavior, and downstream diagnostic quality within structured urologic pathways, in order to better evaluate how early detection strategies translate into clinically meaningful and equitable outcomes.

## 5. Conclusions

In this analysis of two independent survey waves conducted 12 years apart within a non-organized German screening setting, we did not observe an independent study phase effect on awareness of PSA-based early detection in primary multivariable models. Educational attainment consistently emerged as the strongest determinant of both awareness and utilization, while increasing age was primarily associated with utilization. In post-stratified sensitivity analyses re-weighting Study Phase 2 to the Study Phase 1 distribution of age category, educational attainment, and smoking status, study phase was positively associated with utilization. Given the observational design, the limited sample size of the second survey wave, and the dependence on compositional assumptions, this finding should be interpreted as exploratory. Overall, the results emphasize the central role of social gradients in shaping early detection behavior within opportunistic health systems. Future research integrating structured sampling strategies and longitudinal follow-up will be necessary to delineate true temporal changes from compositional effects in decentralized screening environments.

## Figures and Tables

**Table 1:** Participant characteristics by study phase.

		SP1 (n = 755)		SP2 (n = 135)		Total (n = 890)		*p*-Value
		n	Percentage	n	Percentage	n	Percentage	
**Age**	**<35**	29	3.8%	2	1.5%	31	3.5%	0.068
	**35–39**	38	5.0%	7	5.2%	45	5.1%	
	**40–44**	43	5.7%	8	5.9%	51	5.7%	
	**45–49**	61	8.1%	7	5.2%	68	7.6%	
	**50–54**	86	11.4%	15	11.1%	101	11.3%	
	**55–59**	120	15.9%	21	15.6%	141	15.8%	
	**60–64**	70	9.3%	15	11.1%	85	9.6%	
	**65–69**	126	16.7%	22	16.3%	148	16.6%	
	**70–74**	117	15.5%	14	10.4%	131	14.7%	
	**From 75**	64	8.5%	21	15.6%	85	9.6%	
	**Not specified**	1	0.1%	3	2.2%	4	0.4%	
**School education**	**Basic Secondary School Qualification**	199	26.4%	14	10.4%	213	23.9%	0.002
	**Secondary school**	276	36.6%	55	40.7%	331	37.2%	
	**Higher school**	44	5.8%	8	5.9%	52	5.8%	
	**University degree**	230	30.5%	52	38.5%	282	31.7%	
	**Not specified**	6	0.8%	6	4.4%	12	1.3%	
**Employment status**	**Jobseeker**	62	8.2%	5	3.7%	67	7.5%	<0.001
	**Employed**	273	36.2%	7	5.2%	280	31.5%	
	**Self-employed**	37	4.9%	61	45.2%	98	11.0%	
	**Pensioner**	374	49.5%	58	43.0%	432	48.5%	
	**Not specified**	9	1.2%	4	3.0%	13	1.5%	
**Family cancer history**	**No**	395	52.3%	61	45.2%	456	51.2%	0.102
	**One case**	241	31.9%	43	31.9%	284	31.9%	
	**Several cases**	106	14.0%	28	20.7%	134	15.1%	
	**Not specified**	13	1.7%	3	2.2%	16	1.8%	
**Children**	**Yes**	645	85.4%	108	80.0%	753	84.6%	0.019
	**No**	98	13.0%	24	17.8%	122	13.7%	
	**Not specified**	12	1.6%	3	2.2%	15	1.7%	
**Smokers**	**Non-smoker**	366	48.5%	85	63.0%	451	50.7%	<0.001
	**Former smoker**	231	30.6%	18	13.3%	249	28.0%	
	**Smoker**	155	20.5%	29	21.5%	184	20.7%	
	**Not specified**	3	0.4%	3	2.2%	6	0.7%	
**Insurance status**	**Statutory**	700	92.7%	118	87.4%	818	91.9%	0.344
	**Private**	50	6.6%	13	9.6%	63	7.1%	
	**Not specified**	5	0.7%	4	3.0%	9	1.0%	

***Legend:* Baseline sociodemographic and clinical characteristics of study participants stratified by study phase.** Data are presented for Study Phase 1 (2009) and Study Phase 2 (2021). Categorical variables are reported as absolute numbers and percentages. Continuous variables are reported as means with standard deviations. Differences between study phases were assessed using chi-squared tests for categorical variables and independent-samples *t*-tests for continuous variables.

**Table 2:** Factors associated with awareness of PSA-based early detection.

Influencing Factor	Univariable Logistic Regression				Multivariable Logistic Regression			
	Odds Ratio	95% Confidence Interval		*p*-Value	Odds Ratio	95% Confidence Interval		*p*-Value
**Study phase**	1.028	0.699	1.513	0.888	1.021	0.664	1.571	0.924
(Reference: SP1)								
**Age**	1.063	1.005	1.124	0.032	0.990	0.926	1.058	0.766
(Continuous variable)								
**School education**	2.021	1.496	2.730	<0.001	1.709	1.239	2.357	0.001
(Reference: basic secondary school qualification or secondary school certificate)								
**Smoking**	0.553	0.397	0.770	<0.001	0.688	0.479	0.989	0.043
(Reference: non-smoker or former smoker)								
**Paternity**	2.081	1.414	3.064	<0.001	1.939	1.249	3.012	0.003
(Reference: no children)								
**Employment status**	1.829	1.108	3.018	0.018	1.528	0.888	2.631	0.126
(Reference: jobseeker)								
**History of cancer in family**	1.210	0.915	1.599	0.182	1.137	0.844	1.531	0.397
(Reference: no)								
**Insurance**	1.556	0.893	2.711	0.119	1.281	0.711	2.305	0.410
(Reference: statutory)								

***Legend:* Univariable and multivariable logistic regression analyses evaluating factors associated with the dichotomous endpoint awareness of prostate-specific antigen-based early detection in the overall study population.** Study phase (SP1 versus SP2), age (modeled as a continuous variable), educational attainment (basic secondary school qualification or secondary school certificate versus higher school certificate or university degree), smoking status (non-smoker or former smoker versus current smoker), paternity (no children versus children), employment status (jobseeker versus employed, self-employed, or pensioner), family history of cancer (no versus yes), and insurance status (statutory versus private) were included as independent variables. Results are presented as odds ratios with 95% confidence intervals.

**Table 3:** Factors associated with utilization of PSA-based early detection.

Influencing Factor	Univariable Logistic Regression				Multivariable Logistic Regression			
	Odds Ratio	95% Confidence Interval		*p*- Value	Odds Ratio	95% Confidence Interval		*p*- Value
**Study phase**	1.476	1.022	2.131	0.038	1.365	0.885	2.105	0.160
(Reference: SP1)								
**Age**	1.417	1.327	1.513	<0.001	1.391	1.291	1.498	<0.001
(Continuous variable)								
**School education**	1.873	1.422	2.469	<0.001	1.632	1.190	2.239	0.002
(Reference: basic secondary school qualification or secondary school certificate)								
**Smoking**	0.378	0.264	0.541	<0.001	0.608	0.405	0.912	0.016
(Reference: non-smoker or former smoker)								
**Paternity**	2.646	1.718	4.076	<0.001	1.327	0.793	2.239	0.282
(Reference: no children)								
**Employment status**	2.473	1.402	4.362	0.002	0.981	0.525	1.831	0.951
(Reference: jobseeker)								
**History of cancer in family**	1.147	0.0877	1.498	0.316	1.197	0.884	1.622	0.245
(Reference: no)								
**Insurance**	1.085	0.669	1.761	0.741	1.314	0.751	2.299	0.338
(Reference: statutory)								

***Legend:* Univariable and multivariable logistic regression analyses evaluating factors associated with the dichotomous endpoint having already undergone prostate-specific antigen-based early detection in the overall study population.** Independent variables included study phase (SP1 versus SP2), age (modeled as a continuous variable), educational attainment, smoking status, paternity, employment status, family history of cancer, and insurance status. Results are reported as odds ratios with corresponding 95% confidence intervals.

**Table 4:** Factors associated with awareness of PSA-based early detection in men aged 45–69 years.

Influencing Factor	Univariable Logistic Regression				Multivariable Logistic Regression			
	Odds Ratio	95% Confidence Interval		*p*- Value	Odds Ratio	95% Confidence Interval		*p*- Value
**Study phase**	1.287	0.747	2.217	0.363	1.252	0.693	2.262	0.456
(Reference: SP1)								
**Age**	0.987	0.862	1.130	0.845	0.924	0.795	1.075	0.308
(Continuous variable)								
**School education**	1.875	1.258	2.794	0.002	1.717	1.113	2.647	0.014
(Reference: basic secondary school qualification or secondary school certificate)								
**Smoking**	0.525	0.343	0.804	0.003	0.595	0.375	0.945	0.028
(Reference: non-smoker or former smoker)								
**Paternity**	1.665	0.930	2.981	0.086	1.707	0.891	3.271	0.107
(Reference: no children)								
**Employment status**	1.429	0.750	2.722	0.278	1.311	0.664	2.586	0.435
(Reference: jobseeker)								
**History of cancer in family**	1.255	0.864	1.825	0.233	1.113	0.747	1.660	0.599
(Reference: no)								
**Insurance**	1.413	0.710	2.811	0.325	1.025	0.495	2.122	0.946
(Reference: statutory)								

***Legend:* Univariable and multivariable logistic regression analyses evaluating factors associated with awareness of prostate-specific antigen-based early detection in the age-restricted subgroup of men aged 45–69 years.** Independent variables included study phase, age (continuous), educational attainment, smoking status, paternity, employment status, family history of cancer, and insurance status. Results are presented as odds ratios with 95% confidence intervals.

**Table 5:** Factors associated with utilization of PSA-based early detection in men aged 45–69 years.

Influencing Factor	Univariable Logistic Regression				Multivariable Logistic Regression			
	Odds Ratio	95% Confidence Interval		*p*- Value	Odds Ratio	95% Confidence Interval		*p*- Value
**Study phase**	1.350	0.838	2.175	0.217	1.227	0.717	2.098	0.455
(Reference: SP1)								
**Age**	1.528	1.338	1.745	<0.001	1.520	1.315	1.757	<0.001
(Continuous variable)								
**School education**	1.879	1.322	2.670	<0.001	1.590	1.077	2.347	0.020
(Reference: basic secondary school qualification or secondary school certificate)								
**Smoking**	0.469	0.307	0.716	<0.001	0.627	0.393	1.001	0.051
(Reference: non-smoker or former smoker)								
**Paternity**	1.320	0.748	2.330	0.338	0.934	0.484	1.803	0.838
(Reference: no children)								
**Employment status**	1.708	0.901	3.236	0.101	1.073	0.543	2.123	0.839
(Reference: jobseeker)								
**History of cancer in family**	1.284	0.914	1.803	0.150	1.382	0.949	2.014	0.092
(Reference: no)								
**Insurance**	1.153	0.645	2.059	0.632	1.136	0.594	2.170	0.700
(Reference: statutory)								

***Legend:* Univariable and multivariable logistic regression analyses evaluating factors associated with having already undergone prostate-specific antigen-based early detection in men aged 45–69 years.** Independent variables included study phase, age (continuous), educational attainment, smoking status, paternity, employment status, family history of cancer, and insurance status. Results are reported as odds ratios with corresponding 95% confidence intervals.

**Table 6:** Overview of published evidence on determinants of PSA-based early detection.

Author	Study Period	Region	Number of Participants	Question	Factors with a Significant Impact on the Endpoint
Johnson et al. [28]	2010 and 2015	USA	15,372	Had PSA testing	Survey year, Nativity, Region, Age, Education, Martial status, Insurance, Family history, Age, Race/ethnicity
Pickles et al. [32]	2018	Australia	2993	Preference for health care regarding PSA-based ED	Education, Health literacy
Littlejohns et al. [29]	2006–2010	UK	212,039	Had PSA testing	Age, Townsend deprivation score, Region, Family history of cancer, Ethnicity, Employment, Lives with a wife or partner, Smoking, Alcohol intake, Standing high, Private healthcare, Vasectomy, Diabetes (self-reported), Heart disease (self-reported), Hypertension (self-reported), Stroke (self-reported)
Golijanin et al. [27]	2020	USA	56,801	Shared decision-making, Talked about PSA, Had PSA testing	Age, Racial disparities, Shared decision-making, Smoking, Colonoscopy, Sigmoidoscopy, Stool test, Insurance, Regular exercise, Vaccinating
Cohn et al. [21]	2007 and 2012	USA	112,221	Had PSA testing	Survey year, Age, Previous PSA value
Frendl et al. [22]	2000–2014	USA	253,139	Receiving ≥ 1 PSA-test per year	Time period, Age
Nair-Shalliker et al. [31]	2012–2014	Australia	62,765	Had PSA testing	Number of general practitioner consultations, Treatment of benign prostatic hyperplasia, Age, Household income, Living area, Education, Lives with wife or partner, Insurance, Region of birth, Number of medications, Stool test, Overall health, Quality of life, Psychosocial distress, Family history of cancer, Diabetes, Overweight, Alcohol, Physical activity, Urinary bother, Smoking, Erectile dysfunction

***Legend:* Summary of selected studies reporting significant determinants of awareness, utilization, or outcomes related to prostate-specific antigen-based early detection of prostate cancer.** The table provides an overview of study populations, evaluated endpoints, and key associated factors to contextualize the findings of the present study within the existing literature.

## Data Availability

The dataset used and analyzed in this study is available from the corresponding author. The data are not publicly available according to the conditions of the ethics approval and the applicable data protection regulations, the full dataset cannot be made publicly available in an unrestricted repository.

## Appendix A. Appendix Information: Appendix Data Associated with This Article Can Be Found in the Online Version at Doi: XXX

**Table A1:** Checklist for Reporting Of Survey Studies (CROSS).

Section/Topic	Item	Item Description	Reported on Page
**Title and abstract**			
Title and abstract	1a	State the word “survey” along with a commonly used term in title or abstract to introduce the study’s design.	TA2
	1b	Provide an informative summary in the abstract, covering background, objectives, methods, findings/results, interpretation/discussion, and conclusions.	TA2
**Introduction**			
Background	2	Provide a background about the rationale of study, what has been previously done, and why this survey is needed.	TA2
Purpose/aim	3	Identify specific purposes, aims, goals, or objectives of the study.	TA2
**Methods**			
Study design	4	Specify the study design in the methods section with a commonly used term (e.g., cross-sectional or longitudinal).	TA2
	5a	Describe the questionnaire (e.g., number of sections, number of questions, number and names of instruments used).	TA2
Data collection methods	5b	Describe all questionnaire instruments that were used in the survey to measure particular concepts. Report target population, reported validity and reliability information, scoring/classification procedure, and reference links (if any).	TA2
	5c	Provide information on pretesting of the questionnaire, if performed (in the article or in an online supplement). Report the method of pretesting, number of times questionnaire was pre-tested, number and demographics of participants used for pretesting, and the level of similarity of demographics between pre-testing participants and sample population.	TA2
	5d	Questionnaire, if possible, should be fully provided (in the article, or as appendices or as an online supplement).	TA2
Sample characteristics	6a	Describe the study population (i.e., background, locations, eligibility criteria for participant inclusion in survey, exclusion criteria).	TA2
	6b	Describe the sampling techniques used (e.g., single stage or multistage sampling, simple random sampling, stratified sampling, cluster sampling, convenience sampling). Specify the locations of sample participants whenever clustered sampling was applied.	TA2
	6c	Provide information on sample size, along with details of sample size calculation.	TA2
	6d	Describe how representative the sample is of the study population (or target population, if possible), particularly for population-based surveys.	TA2
Survey administration	7a	Provide information on modes of questionnaire administration, including the type and number of contacts and the location where the survey was conducted (e.g., outpatient room or by use of online tools, such as SurveyMonkey).	TA2
	7b	Provide information of survey’s time frame, such as periods of recruitment, exposure, and follow-up days.	TA2
	7c	Provide information on the entry process: –>For non-web-based surveys, provide approaches to minimize human error in data entry. –>For web-based surveys, provide approaches to prevent “multiple participation” of participants.	TA2
Study preparation	8	Describe any preparation process before conducting the survey (e.g., interviewers’ training process, advertising the survey).	TA2
Ethical considerations	9a	Provide information on ethical approval for the survey if obtained, including informed consent, institutional review board approval, Helsinki declaration, and good clinical practice (GCP) declaration (as appropriate).	TA2
	9b	Provide information about survey anonymity and confidentiality and describe what mechanisms were used to protect unauthorized access.	TA2
Statistical analysis	10a	Describe statistical methods and analytical approach. Report the statistical software that was used for data analysis.	TA2
	10b	Report any modification of variables used in the analysis, along with reference (if available).	TA2
	10c	Report details about how missing data were handled. Include rate of missing items, missing data mechanism (i.e., missing completely at random [MCAR], missing at random [MAR], or missing not at random [MNAR]) and methods used to deal with missing data (e.g., multiple imputation).	TA2
	10d	State how non-response error was addressed.	TA2
	10e	For longitudinal surveys, state how loss to follow-up was addressed.	TA2
	10f	Indicate whether any methods such as weighting of items or propensity scores have been used to adjust for non-representativeness of the sample.	TA2
	10g	Describe any pre-specified subgroup analysis conducted.	TA2
**Results**			
Respondent characteristics	11a	Report numbers of individuals at each stage of the study. Consider using a flow diagram, if possible.	TA2
	11b	Provide reasons for non-participation at each stage, if possible.	TA2
	11c	Report response rate; present the definition of response rate or the formula used to calculate response rate.	TA2
	11d	Provide information to define how unique visitors are determined. Report number of unique visitors along with relevant proportions (e.g., view proportion, participation proportion, completion proportion).	TA2
Descriptive results	12	Provide characteristics of study participants, as well as information on potential confounders and assessed outcomes.	TA2
Main findings	13a	Give unadjusted estimates and, if applicable, confounder-adjusted estimates along with 95% confidence intervals and *p*-values.	TA2
	13b	For multivariable analysis, provide information on the model building process, model fit statistics, and model assumptions (as appropriate).	TA2
	13c	Provide details about any pre-specified subgroup analysis performed. If there are considerable amounts of missing data, report sensitivity analyses comparing the results of complete cases with that of the imputed dataset (if possible).	TA2
**Discussion**			
Limitations	14	Discuss the limitations of the study, considering sources of potential biases and imprecisions, such as non-representativeness of sample, study design, and important uncontrolled confounders.	TA2
Interpretations	15	Give a cautious overall interpretation of results, based on potential biases and imprecisions, and suggest areas for future research.	TA2
Generalizability	16	Discuss the external validity of the results.	TA2
**Other sections**			
Role of funding source	17	State whether any funding organization has had any roles in the survey’s design, implementation, and analysis.	TA2
Conflict of interest	18	Declare any potential conflict of interest.	TA2
Acknowledgements	19	Provide names of organizations/persons that are acknowledged along with their contribution to the research.	TA2

*Legend:* TA2, Table A2.

**Table A2:** Locations of all 19 items of the CROSS checklist within the manuscript.

Section/Topic	Item	Reported on Page or Explanation for Non-Inclusion
**Title and abstract**		
Title and abstract	1a	1
Title and abstract	1b	1
**Introduction**		
Background	2	2
Purpose/Aim	3	3
**Methods**		
Study design	4	4
Data collection methods	5a	4
Data collection methods	5b	4
Data collection methods	5c	4
Data collection methods	5d	Figure A1 and Figure A2
Sample characteristics	6a	4
Sample characteristics	6b	4
Sample characteristics	6c	The KABOT study fundamentally serves as a hypothesis-generating investigation, thereby lacking precedent studies from other research groups to validate a reliable effect size necessary for biometric sample size calculation. Furthermore, the study employed a highly structured form of cluster sampling in recruiting the study cohort, aiming to motivate the inclusion of approximately 50 patients from all general practitioners within clearly defined regions. This approach, too, posed challenges in conducting a biometric sample size calculation.
Sample characteristics	6d	Throughout both study periods, 29 general practitioners in the Berlin (urban) and Brandenburg (rural) regions participated, resulting in the distribution of 1450 questionnaires to eligible male patients. The overall response rate was 61.4% (890/1450), with 72.0% in Study Phase 1 (SP1) and 33.8% in Study Phase 2 (SP2). The ethics approval covered this anonymous, non-interventional survey; participation was voluntary and implied by the return of a completed questionnaire. No data were collected from non-responders (n = 560). Accordingly, we were unable to compare characteristics of responders and non-responders and cannot formally assess the representativeness of the responding sample.
Survey administration	7a	4
Survey administration	7b	4
Survey administration	7c	The transfer of the entire dataset from the returned questionnaires into the SPSS table used for statistical exploration was independently performed by two members of the research team (KPB and TV). Subsequently, various data entries were identified and cross-checked with patient information in the original questionnaires. This process significantly mitigated the presence of erroneous entries in the final SPSS table utilized for statistical analysis.
Study preparation	8	4
Ethical considerations	9a	4
Ethical considerations	9b	4
Statistical analysis	10a	6
Statistical analysis	10b	No modification of study variables was conducted.
Statistical analysis	10c	5
Statistical analysis	10d	5
Statistical analysis	10e	For these analyses, this statement does not apply to the KABOT study.
Statistical analysis	10f	For these analyses, this statement does not apply to the KABOT study.
Statistical analysis	10g	Post-stratification sensitivity analyses were conducted to assess the robustness of temporal associations to compositional differences between survey waves. In addition, the validity of the presented results was examined concerning different age groups and also in relation to the patient’s insurance status, which was an identified study objective (pre-specified subgroup analysis). 5
**Results**		
Respondent characteristics	11a	6
Respondent characteristics	11b	Due to the design of the KABOT study, there are two types of non-responders: 1. The addressed general practitioners, of whom only 29 out of a total of 205 (14.2%) participated in the study despite reminders for their involvement. This is largely attributable to the insufficient density of general practices in the federal state of Brandenburg, where many general practitioners routinely work over 80 h per week. The extensive workload simply leaves many colleagues with no spare time to support academic research. This observation was undoubtedly exacerbated during Study Phase 2 (SP2) due to the burdens of the COVID-19 pandemic. While 38.2% of the initially addressed general practitioners participated in SP1, this figure dropped to a mere 5.3% in SP2. 2. The non-participation of 38.6% of male patients, on the other hand, falls within the expected range as known from other questionnaire-based studies involving patients. The reasons for this are diverse and have been analyzed in numerous studies on the subject.
Respondent characteristics	11c	6
Respondent characteristics	11d	The response to this item has been documented in Figure 1. Here is a brief summary: 1450 questionnaires were distributed by the 29 general practitioners to 1450 male patients, which were subsequently viewed by these patients. Ultimately, out of these initial 1450 patients, 890 patients (61.4%) opted to participate in the study and returned the completed questionnaires.
Descriptive results	12	Table 1
Main findings	13a	The descriptive analyses presented in the KABOT study were unadjusted.
Main findings	13b	For the analyses presented in the KABOT study, this statement does not apply.
Main findings	13c	The handling of non-respondent patients was previously addressed in our response to Item 6d, and the execution of subgroup analyses (as a substitute for sensitivity analyses) was detailed in our response to Item 10g. Among the 890 patients constituting the study group, some of the returned questionnaires were not fully completed for every question (the criterion for questionnaire inclusion was >95% completeness of responses). In Table 1, all missing data in individual questions were labeled as ‘not specified’, with missing responses to individual study endpoints ranging between 1 and 12 (equivalent to 0.11% to 1.3%).
**Discussion**		
Limitations	14	15
Interpretations	15	12 to 15
Generalizability	16	11 to 15
**Other sections**		
Role of funding source	17	16
Conflict of interest	18	16
Acknowledgements	19	There are no additional doctors or institutions beyond those listed in the author group who require acknowledgment. A comprehensive acknowledgment was extended to the general practitioners participating over the 12-year study period, as well as to the patients.

**Table A3:** Item-level missingness.

Influencing Factor	Study Phase 1		Study Phase 2	
	n	Percentage	N	Percentage
**Age**	1	0.1	2	1.5
**School education**	6	0.8	6	4.4
**Smoking**	3	0.4	3	2.2
**Paternity**	12	1.6	3	2.2
**Employment status**	9	1.2	4	3.0
**History of cancer in family**	13	1.7	3	2.2
**Insurance**	3	0.4	4	3.0

**Table A4:** Factors associated with awareness of PSA-based early detection with educational attainment coded in four levels and smoking status coded as non-smoker, former and current smoker.

Influencing Factor	Multivariable Logistic Regression			
	Odds Ratio	95% Confidence Interval		*p*-Value
**Study phase**	0.962	0.619	1.497	0.865
(Reference: SP1)				
**Age**	1.011	0.937	1.091	0.784
(Continuous variable)				
**School education**				
Secondary school	1.724	1.130	2.632	0.012
Higher school	1.134	0.568	2.265	0.722
University degree	2.893	1.886	4.436	<0.001
(Reference: Basic secondary school)				
**Smoking**				
Former smoker	1.224	0.850	1.763	0.277
Smoker	0.716	0.486	1.055	0.091
(Reference: Non-smoker)				
**Paternity**	1.787	1.145	2.789	0.011
(Reference: no children)				
**Employment status**	1.489	0.860	2.577	0.355
(Reference: jobseeker)				
**History of cancer in family**	1.070	0.791	1.447	0.660
(Reference: no)				
**Insurance**	1.291	0.713	2.339	0.399
(Reference: statutory)				

**Table A5:** Factors associated with awareness of PSA-based early detection in a post-stratification sensitivity analysis aligning Study Phase 2 to the Study Phase 1 distribution with respect to age, educational attainment, and smoking status.

Influencing Factor	Multivariable Logistic Regression			
	Odds Ratio	95% Confidence Interval		*p*-Value
**Study phase**	1.143	0.739	1.769	0.548
(Reference: SP1)				
**Age**	0.999	0.934	1.067	0.969
(Continuous variable)				
**School education**	1.722	1.243	2.386	0.001
(Reference: basic secondary school qualification or secondary school certificate)				
**Smoking**	0.694	0.483	0.998	0.049
(Reference: non-smoker or former smoker)				
**Paternity**	1.969	1.271	3.051	0.002
(Reference: no children)				
**Employment status**	1.528	0.890	2.625	0.124
(Reference: jobseeker)				
**History of cancer in family**	1.104	0.819	1.488	0.516
(Reference: no)				
**Insurance**	1.310	0.722	2.375	0.374
(Reference: statutory)				

**Table A6:** Factors associated with utilization of PSA-based early detection with educational attainment coded in four levels and smoking status coded as non-smoker, former and current smoker.

Influencing Factor	Multivariable Logistic Regression			
	Odds Ratio	95% Confidence Interval		*p*-Value
**Study phase**	1.152	0.730	1.817	0.544
(Reference: SP1)				
**Age**	1.493	1.367	1.631	<0.001
(Continuous variable)				
**School education**				
Secondary school	2.855	1.791	4.550	<0.001
Higher school	1.181	0.496	2.814	0.707
University degree	3.458	2.239	5.339	<0.001
(Reference: Basic secondary school)				
**Smoking**				
Former smoker	0.899	0.629	1.287	0.562
Smoker	0.574	0.374	0.883	0.011
(Reference: Non-smoker)				
**Paternity**	1.169	0.689	1.981	0.563
(Reference: no children)				
**Employment status**	1.001	0.528	1.901	0.996
(Reference: jobseeker)				
**History of cancer in family**	1.130	0.829	1.542	0.439
(Reference: no)				
**Insurance**	1.297	0.713	2.304	0.374
(Reference: statutory)				

**Table A7:** Factors associated with utilization of PSA-based early detection in a post-stratification sensitivity analysis aligning Study Phase 2 to the Study Phase 1 distribution with respect to age, educational attainment, and smoking status.

Influencing Factor	Multivariable Logistic Regression			
	Odds Ratio	95% Confidence Interval		*p*-Value
**Study phase**	1.619	1.043	2.513	0.032
(Reference: SP1)				
**Age**	1.404	1.303	1.513	<0.001
(Continuous variable)				
**School education**	1.635	1.188	2.251	0.003
(Reference: basic secondary school qualification or secondary school certificate)				
**Smoking**	0.627	0.418	0.941	0.024
(Reference: non-smoker or former smoker)				
**Paternity**	1.328	0.796	2.215	0.278
(Reference: no children)				
**Employment status**	0.978	0.524	1.823	0.943
(Reference: jobseeker)				
**History of cancer in family**	1.168	0.861	1.585	0.318
(Reference: no)				
**Insurance**	1.329	0.755	2.340	0.325
(Reference: statutory)				

**Table A8:** Practice-level cluster-robust standard errors in multivariable models.

Dependent Variable	Odds Ratio	95% Confidence Interval		*p*-Value
**Awareness of PSA**	0.53	0.44	0.64	<0.001
**Utilization of PSA**	1.25	0.91	1.72	0.162

**Table A9:** Evaluation of multicollinearity using variance inflation factors for significant influencing factors of awareness and utilization of PSA-based early detection.

Endpoint	Influencing Factor	Variance Inflation Factor
**Awareness**	**School education**	1.079
	**Smoking**	1.101
	**Paternity**	1.152
**Utilization**	**Age**	1.256
	**School education**	1.079
	**Smoking**	1.101

Note: Variance inflation factor levels were low (between 1.079 and 1.256) and remained below established thresholds, excluding relevant multicollinearity.

**Table A10:** Receiver Operating Characteristics (ROC) analysis for awareness and utilization of PSA-based early detection.

Endpoint	AUC	Standard Error	95% Confidence Interval		*p*-Value
**Awareness**	0.631	0.02	0.591	0.670	<0.001
**Utilization**	0.729	0.0017	0.695	0.763	<0.001
